# Long-term survival after surgical treatment for post-infarction mechanical complications: results from the Caution study

**DOI:** 10.1093/ehjqcco/qcae010

**Published:** 2024-02-07

**Authors:** Matteo Matteucci, Daniele Ronco, Mariusz Kowalewski, Giulio Massimi, Michele De Bonis, Francesco Formica, Federica Jiritano, Thierry Folliguet, Nikolaos Bonaros, Sandro Sponga, Piotr Suwalski, Andrea De Martino, Theodor Fischlein, Giovanni Troise, Guglielmo Actis Dato, Filiberto Giuseppe Serraino, Shabir Hussain Shah, Roberto Scrofani, Jurij Matija Kalisnik, Andrea Colli, Claudio Francesco Russo, Marco Ranucci, Matteo Pettinari, Adam Kowalowka, Matthias Thielmann, Bart Meyns, Fareed Khouqeer, Jean-Francois Obadia, Udo Boeken, Caterina Simon, Shiho Naito, Andrea Musazzi, Roberto Lorusso

**Affiliations:** Department of Cardiothoracic Surgery, Heart and Vascular Centre, Maastricht University Medical Centre, Maastricht, The Netherlands; Department of Medicine and Surgery, Circolo Hospital, University of Insubria, Varese, Italy; Thoracic Research Centre, Collegium Medicum Nicolaus Copernicus University, Innovative Medical Forum, Bydgoszcz, Poland; Department of Cardiothoracic Surgery, Heart and Vascular Centre, Maastricht University Medical Centre, Maastricht, The Netherlands; Thoracic Research Centre, Collegium Medicum Nicolaus Copernicus University, Innovative Medical Forum, Bydgoszcz, Poland; Cardiac Surgery Unit, Cardio-Thoraco-Vascular Department, Niguarda Hospital, Milan, Italy; Thoracic Research Centre, Collegium Medicum Nicolaus Copernicus University, Innovative Medical Forum, Bydgoszcz, Poland; Department of Cardiac Surgery and Transplantology, National Medical Institute of the Ministry of Interior, Warsaw, Poland; Department of Cardiothoracic Surgery, Heart and Vascular Centre, Maastricht University Medical Centre, Maastricht, The Netherlands; Thoracic Research Centre, Collegium Medicum Nicolaus Copernicus University, Innovative Medical Forum, Bydgoszcz, Poland; Cardiothoracic Surgery Department, San Raffaele University Hospital, Milan, Italy; Department of Medicine and Surgery, University of Parma, Cardiac Surgery Unit, University Hospital of Parma, Parma, Italy; Department of Cardiothoracic Surgery, Heart and Vascular Centre, Maastricht University Medical Centre, Maastricht, The Netherlands; Thoracic Research Centre, Collegium Medicum Nicolaus Copernicus University, Innovative Medical Forum, Bydgoszcz, Poland; Department of Experimental and Clinical Medicine, “Magna Graecia” University of Catanzaro, Catanzaro, Italy; Department of Cardio-Thoracic Surgery, University Hospital Henri-Mondor, Créteil, Paris, France; Department of Cardiac Surgery, Medical University of Innsbruck, Innsbruck, Austria; Cardiothoracic Department, University Hospital of Udine, Udine, Italy; Department of Cardiac Surgery and Transplantology, National Medical Institute of the Ministry of Interior, Warsaw, Poland; Section of Cardiac Surgery, University Hospital, Pisa, Italy; Department of Cardiac Surgery, Cardiovascular Center, Klinikum Nürnberg, Paracelsus Medical University, Nuremberg, Germany; Cardiac Surgery Unit, Poliambulanza Foundation Hospital, Brescia, Italy; Cardiac Surgery Department, Mauriziano Hospital, Turin, Italy; Department of Experimental and Clinical Medicine, “Magna Graecia” University of Catanzaro, Catanzaro, Italy; Cardiovascular and Thoracic Surgery Department, King Fahad Medical City, Riyadh, Saudi Arabia; Cardiac Surgery Unit, Fondazione Ospedale Maggiore Policlinico IRCCS Cà Granda, University of Milan, Milan, Italy; Department of Cardiac Surgery, Cardiovascular Center, Klinikum Nürnberg, Paracelsus Medical University, Nuremberg, Germany; Section of Cardiac Surgery, University Hospital, Pisa, Italy; Cardiac Surgery Unit, Cardio-Thoraco-Vascular Department, Niguarda Hospital, Milan, Italy; Department of Cardiovascular Anesthesia and Intensive Care, IRCCS Policlinico San Donato, San Donato Milanese, Italy; Department of Cardiovascular Surgery, Ziekenhuis Oost-Limburg, Genk, Belgium; Department of Cardiac Surgery, Medical University of Silesia, Katowice, Poland; Department of Thoracic and Cardiovascular Surgery, West-German Heart Center, University of Duisburg-Essen, Essen, Germany; Department of Cardiac Surgery, University Hospitals Leuven, Leuven, Belgium; Department of Cardiac Surgery, King Faisal Specialist Hospital and Research Center, Riyadh, Saudi Arabia; Department of Cardiac Surgery, Louis Pradel Cardiologic Hospital, Lyon, France; Department of Cardiovascular Surgery, University Hospital Düsseldorf, Heinrich Heine University, Düsseldorf, Germany; Cardiovascular and Transplant Department, Papa Giovanni XXIII Hospital, Bergamo, Italy; Department of Cardiovascular Surgery, University Heart & Vascular Center Hamburg, Hamburg, Germany; Department of Medicine and Surgery, Circolo Hospital, University of Insubria, Varese, Italy; Department of Cardiothoracic Surgery, Heart and Vascular Centre, Maastricht University Medical Centre, Maastricht, The Netherlands; Cardiovascular Research Institute Maastricht, Maastricht, The Netherlands

**Keywords:** Mechanical complications, Acute myocardial infarction, Surgical treatment, Ventricular septal rupture, Free-wall rupture, Papillary muscle rupture

## Abstract

**Aims:**

Mechanical complications (MCs) are rare but potentially fatal sequelae of acute myocardial infarction (AMI). Surgery, though challenging, is considered the treatment of choice. The authors sought to study the early and long-term results of patients undergoing surgical treatment for post-AMI MCs.

**Methods and results:**

Patients who underwent surgical treatment for post-infarction MCs between 2001 through 2019 in 27 centres worldwide were retrieved from the database of the CAUTION study. In-hospital and long-term mortality were the primary outcomes. Cox proportional hazards regression models were used to determine independent factors associated with overall mortality. The study included 720 patients. The median age was 70.0 [62.0–77.0] years, with a male predominance (64.6%). The most common MC encountered was ventricular septal rupture (VSR) (59.4%). Cardiogenic shock was seen on presentation in 56.1% of patients. In-hospital mortality rate was 37.4%; in more than 50% of cases, the cause of death was low cardiac output syndrome (LCOS). Late mortality occurred in 133 patients, with a median follow-up of 4.4 [1.0–8.6] years. Overall survival at 1, 5, and 10 years was 54.0, 48.1, and 41.0%, respectively. Older age (*P* < 0.001) and post-operative LCOS (*P* < 0.001) were independent predictors of overall mortality. For hospital survivors, 10-year survival was 65.7% and was significantly higher for patients with VSR than those with papillary muscle rupture (long-rank *P* = 0.022).

**Conclusion:**

Contemporary data from a multicentre cohort study show that surgical treatment for post-AMI MCs continues to be associated with high in-hospital mortality rates. However, long-term survival in patients surviving the immediate post-operative period is encouraging.

## Graphical Abstract

**Graphical Abstract:**
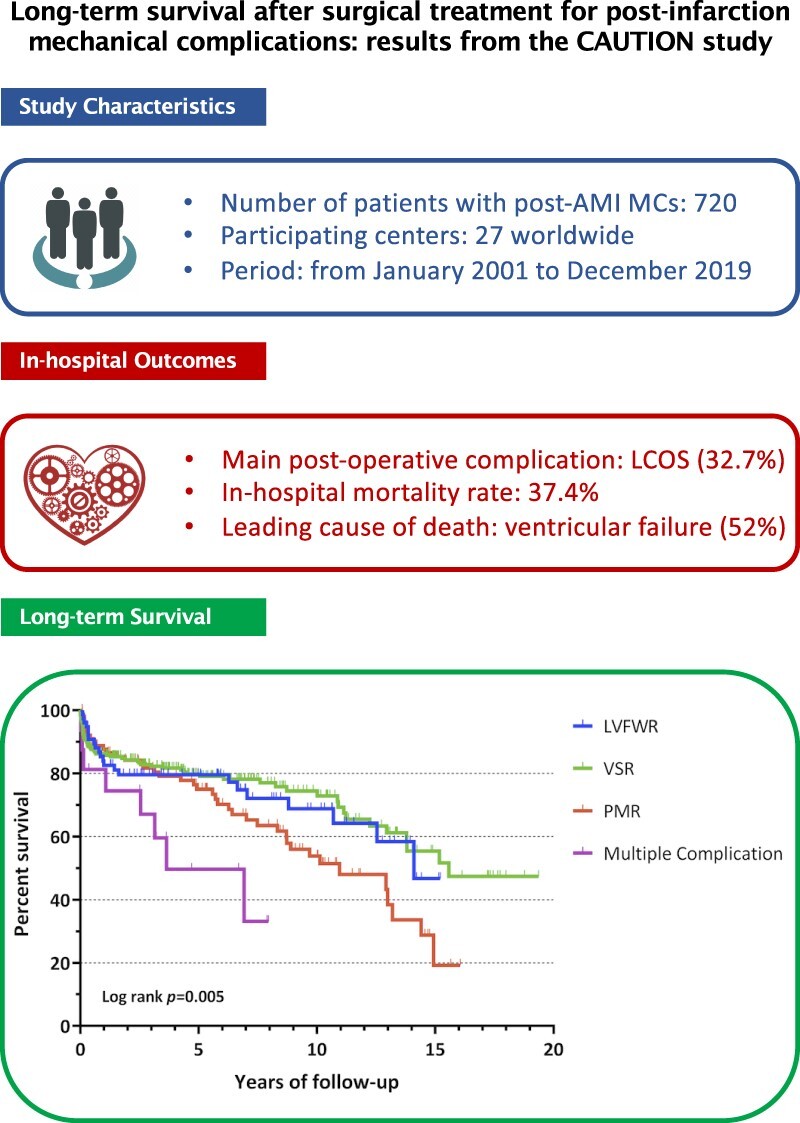

Key learning points What is already known

- Mechanical complications are rare but potentially lethal sequelae of acute myocardial infarction.
- Surgical treatment is considered the standard of care; however, given the rarity of these conditions, the results after surgery are not well established.

What this study adds

- Surgical treatment for post-infarction mechanical complications is associated with high in-hospital mortality (∼37%).
- Long-term survival for patients surviving the immediate post-operative period is encouraging (10-year survival of ∼66%).

## Introduction

Left ventricular free-wall rupture (LVFWR), ventricular septal rupture (VSR), and papillary muscle rupture (PMR) represent the most common mechanical complications (MCs) of acute myocardial infarction (AMI).^1^ Over the last decades, advances in acute reperfusion strategies for AMI have led to a decline in the incidence of MCs.^2,3^ However, patients with large infarcts or those who do not receive appropriate early revascularization remain at risk of developing these complications. Moreover, during the recent COVID-19 pandemic, there has been a new surge in the number of MCs from ST-elevation myocardial infarction (STEMI).^4^

Though challenging and associated with very high morbidity and mortality rates, surgery is still considered the treatment of choice for patients who develop post-AMI MCs, especially given the almost inevitably fatal outcome associated with conservative management.^5^ However, because of the rarity of MCs, the current evidence is mainly based on small series and single-centre experiences, and little is known about the surgical results in the contemporary era of advanced techniques and therapies. Furthermore, given the high in-hospital mortality, most studies focus on short-term outcomes, and the evidence regarding the late consequences of post-AMI MCs for patients surviving the early post-operative phase has not been elucidated yet.

In the present study, therefore, we sought to evaluate the in-hospital outcomes and long-term survival of patients undergoing cardiac surgery for post-infarction MCs using a multicentre international registry.

## Methods

### Trial design and patients

**Figure 1:**
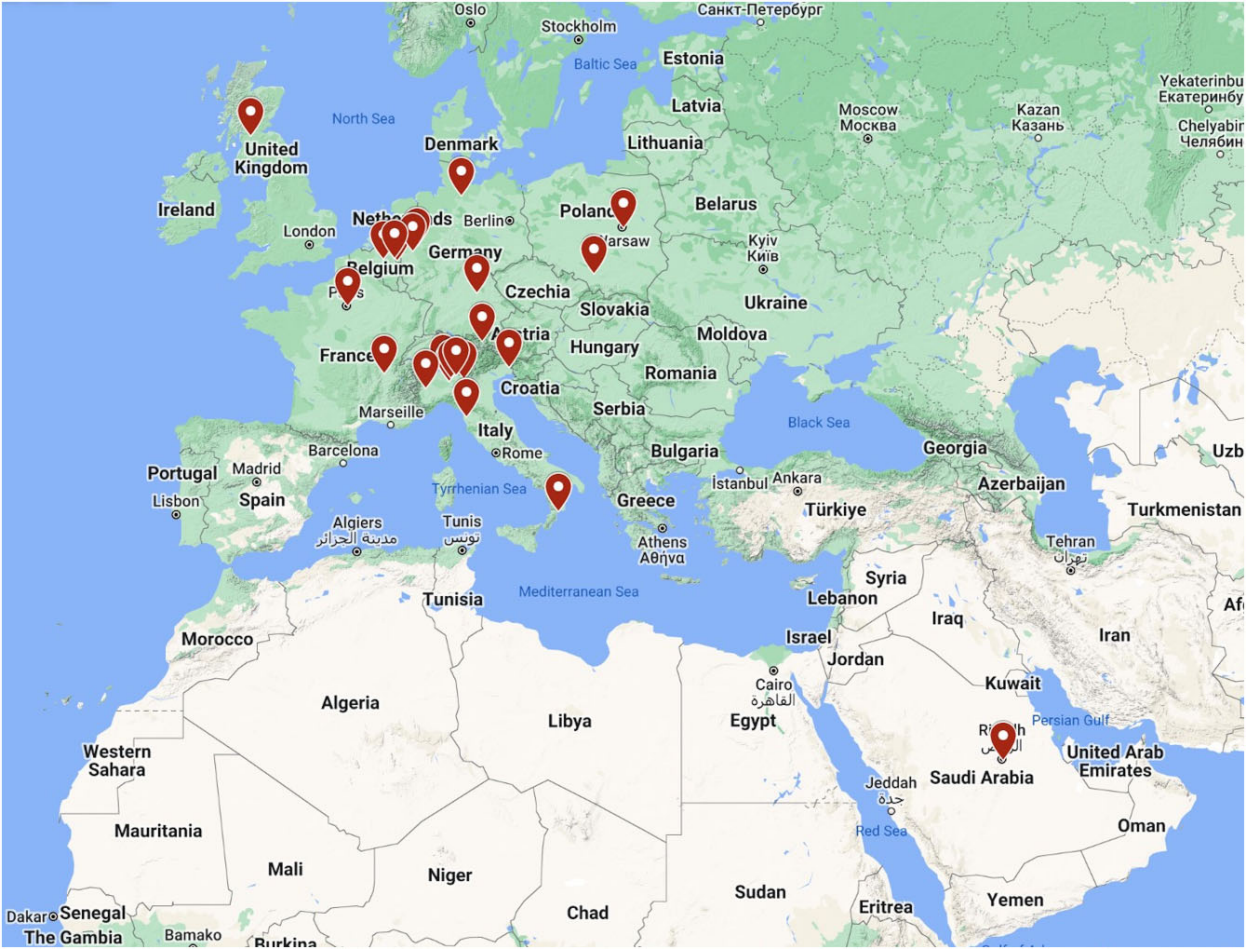
Distribution of the centers involved in the study.

CAUTION study (meChanical complicAtions of acUte myocardial infarcTion: an InternatiOnal multiceNter cohort study; NCT03848429) is a retrospective, international, multicentre trial aimed at evaluating the post-operative outcomes and survival of patients undergoing cardiac surgery for post-AMI MCs between January 2001 and December 2019. Data were collected from 27 different centres worldwide, belonging to 9 countries (*Figure 1*). The study protocol was approved by the ethical committee of the promoting centre (Maastricht University Medical Centre, Maastricht, the Netherlands; METC 2018–0924) and authorized by the local ethical committees of each participating centre. The trial was conducted in accordance with the guidelines of the declaration of Helsinki for patient data utilization and evaluation.

For the purpose of our study, from the CAUTION study database, we recruited all adult patients (aged >18 years) who underwent surgical treatment for post-infarction MCs and for whom data about the long-term survival were available. Exclusion criteria included patients who underwent medical/conservative or percutaneous treatment for MCs and patients who underwent surgery for heart rupture unrelated to AMI (e.g. chest trauma). Criteria for indications of surgical repair across different centres have neither been collected nor analysed.

### Definitions and outcome measures

Cardiogenic shock (CS) was defined as persistent hypotension (systolic blood pressure <90 mmHg) with a reduction in cardiac index (<1.8 L/min/m^2^) despite maximal treatment. Critical pre-operative state included patients with either CS or impending haemodynamic instability, defined as radiological signs of acute pulmonary oedema, echocardiographic signs of failing univentricular or biventricular function, or persistent or worsening signs and symptoms of acute heart failure.

In-hospital (or early) mortality was defined as all-cause death that occurred within 30 days from the intervention or during the hospitalization related to the operation, while late mortality >30 days or after hospital discharge. Intra-operative mortality was considered a death occurring during the surgical procedure.

The primary endpoints were in-hospital and long-term mortality. Additional outcomes included causes of in-hospital death and identification of prognostic factors associated with overall, early, and late mortality.

### Statistical analysis

Continuous variables were tested for normality distribution with the Shapiro–Wilk test and were summarized as mean ± standard deviation (variables not violating the normality assumption) or median with interquartile range (IQR) (variables violating the normality assumption). Categorical variables were reported as frequencies with valid percentages. An analysis of patterns was performed. Considering that a very limited number of variables had more than 10% of missing values (and anyway, almost all <30%) analysis was carried out without multiple imputations for missing values.

Differences between groups were assessed. Categorical variables were analysed individually (univariate analysis) with the Chi-square test or with the Fisher's exact test, as appropriate. Continuous variables were compared with the Student's t-test or the Mann–Whitney *U*-test, as appropriate. Variables of clinical interest that achieved a *P*-value <0.05 at the univariate analysis were tested for multicollinearity and then entered into three multivariable Cox regression models to identify independent predictors of early and overall mortality. Results were presented as a Hazard Ratio (HR) with a 95% confidence interval (CI).

Survival curves were constructed with the Kaplan–Meier method for both the whole population and hospital survivors only, with subgroup analyses according to type of MCs, and were compared using the long-rank test.

All the statistical analyses were conducted using SPSS 26.0 (IBM Corp. Release 2019, IBM SPSS Statistics for Windows, Version 26.0, Armonk, NY, USA) and GraphPad Prism 8 (GraphPad Software for Windows, Version 8.0.1, San Diego, CA, USA). A two-tailed *P*-value <0.05 was considered statistically significant.

## Results

### Patient characteristics

After excluding cases with missing data on follow-up (*n* = 63), a total of 720 patients constituted the study cohort. Baseline characteristics are presented in *Table 1*. The median age at admission was 70.0 (IQR: 62.0–77.0) years, with a prevalence of male patients of 64.6%. Almost 90% of subjects on admission had an ECG pattern of STEMI, mostly inferior. Hypertension was the most common comorbidity.

**Table 1:** Baseline characteristics of patients according to overall survival

Variable	Patients (*n* = 720)	Alive (*n* = 318)	Dead (*n* = 402)	*P*-value
Age (years)	70.0 (62.0–77.0)	67.0 (60.0–73.3)	72.0 (64.0–78.0)	<0.001
Sex (male)	465 (64.6)	217 (68.2)	248 (61.7)	0.068
Hypertension	445 (61.8)	198 (62.3)	247 (61.4)	0.822
Smoking	270 (37.5)	131 (41.2)	139 (34.6)	0.069
Diabetes mellitus	162 (22.5)	63 (19.8)	99 (24.6)	0.124
Chronic kidney disease	91 (12.6)	29 (9.1)	62 (15.4)	0.011
Dyslipidaemia	240 (33.3)	122 (38.4)	118 (29.4)	0.011
ECG pattern STEMI NSTEMI	606 (86.9) 91 (13.1)	268 (86.5) 42 (13.5)	338 (87.3) 49 (12.7)	0.730
AMI localization Septal/anterior Lateral Inferior	257 (41.7) 48 (7.8) 312 (50.6)	114 (41.5) 24 (8.7) 137 (49.8)	143 (41.8) 24 (7.0) 175 (51.2)	0.729
Pre-operative stability Stable Unstable	235 (32.6) 485 (67.4)	132 (41.5) 186 (58.5)	103 (25.6) 299 (74.4)	<0.001
Cardiogenic shock	404 (56.1)	141 (44.3)	263 (65.4)	<0.001
Cardiac arrest	73 (10.1)	15 (4.7)	58 (14.4)	<0.001
Cardiac tamponade	119 (16.5)	48 (15.1)	71 (17.7)	0.357
Pre-operative IABP	393 (54.6)	159 (50.0)	234 (58.2)	0.028
Pre-operative ECMO	63 (8.8)	18 (5.7)	45 (11.2)	0.009
Pre-operative LVEF (%)	45.0 (35.0–50.0)	45.0 (39.0–52.0)	40.0 (30.0–50.0)	0.001
Coronary angiography	600 (83.3)	272 (85.5)	328 (81.6)	0.159
PCI	205 (28.5)	81 (25.6)	124 (30.8)	0.119
Previous thrombolysis	44 (6.1)	15 (4.7)	29 (7.2)	0.165
Hours from MC diagnosis to surgery	24.0 (3.0–108.0)	42.0 (4.0–168.0)	11.0 (3.0–72.0)	<0.001

Values are median (interquartile range) or *n* (%).

STEMI, ST-elevation myocardial infarction; NSTEMI, non-ST-elevation myocardial infarction; AMI, acute myocardial infarction; IABP, intra-aortic balloon pump; ECMO, extracorporeal membrane oxygenation; LVEF, left ventricular ejection fraction; PCI, percutaneous coronary intervention.

More than two thirds of patients were haemodynamically unstable at presentation, and half of the whole population met the criteria of CS. Thus, more than 50% of subjects received preoperative implantation of an intra-aortic balloon pump (IABP), and 8.8% also needed extracorporeal membrane oxygenation (ECMO) support.

Before surgery, 83.3% of patients underwent coronary angiography, but PCI was performed in less than 30% of them and thrombolysis in about 6%. VSR was the most common MC identified (*n* = 428), followed by PMR (*n* = 139) and LVFWR (*n* = 120); coexistence of multiple MCs was observed in 33 subjects. The median time from cardiac rupture diagnosis to surgery was 24.0 (IQR: 3.0–108.0) h, highly variable according to different types of MCs.

### Perioperative information

Perioperative characteristics are summarized in *Table 2*. Anterior VSR, oozing LVFWR, and complete PMR were the most frequent types of ruptures encountered. The vast majority of patients, mostly VSR and PMR, were treated on cardiopulmonary bypass (CPB) with an aortic cross-clamp. On the contrary, out of 150 patients with LVFWR, 75 were managed with beating hearts, and 55 did not require CPB support. Concomitant coronary artery bypass grafting (CABG) was performed in slightly less than half of the individuals.

**Table 2:** Perioperative and operative data of patients according to overall survival

Variable	Patients (*n* = 720)	Alive (*n* = 318)	Dead (*n* = 402)	*P*-value
Type of rupture^c^				
Anterior VSR Posterior VSR	229 (54.5) 191 (45.5)	109 (59.2) 75 (40.8)	120 (50.8) 116 (49.2)	0.087
Oozing LVFWR Blowout LVFWR	81 (57.0) 61 (43.0)	39 (67.2) 19 (32.8)	42 (50.0) 42 (50.0)	0.041
Partial PMR Complete PMR	37 (39.8) 57 (60.2)	16 (40.0) 24 (60.0)	21 (39.6) 32 (60.4)	0.971
Repair technique or procedure to treat post-AMI MC^c^				
Infarct exclusion Other VSR repair	81 (18.8) 350 (81.2)	43 (22.8) 146 (77.2)	38 (15.7) 204 (84.3)	0.063
Sutured LVFWR repair Sutureless LVFWR repair	96 (64.9) 52 (35.1)	32 (52.5) 29 (47.5)	64 (73.6) 23 (26.4)	0.008
MV replacement MV repair	118 (78.7) 32 (21.3)	53 (79.1) 14 (20.9)	65 (78.3) 18 (21.7)	0.906
Pericardiocentesis (LVFWR)	30 (20.0)	9 (14.8)	21 (23.3)	0.195
CPB	665 (92.4)	293 (92.1)	372 (92.5)	0.841
ACC	640 (88.9)	287 (90.3)	353 (87.8)	0.301
CPB time (min)	133.0 (98.0–177.0)	124.0 (90.0–164.0)	138.0 (103.0–181.5)	0.002
ACC time (min)	84.0 (65.0–112.5)	80.0 (60.0–108.5)	89.0 (68.3–115.0)	0.008
Concomitant CABG	340 (47.2)	156 (49.1)	184 (45.8)	0.381
Post-operative IABP	358 (56.7)	129 (48.5)	229 (62.7)	<0.001
Post-operative ECMO	82 (12.3)	24 (8.0)	58 (15.8)	0.002
Post-operative inotropes	549 (80.0)	242 (76.1)	315 (81.0)	0.115
Rethoracotomy for bleeding^a^	82 (12.0)	23 (7.2)	59 (16.0)	<0.001
Reoperation^a^	62 (9.0)	24 (7.5)	38 (10.3)	0.206
Rupture recurrence^a^ Requiring reintervention^a^	77 (11.2%) 32 (4.7)	24 (7.5%) 12 (3.8)	48 (13.0%) 20 (5.4)	0.019 0.304
Intensive care unit stay^a^ (h)	144.0 (71.8–271.5)	120.0 (56.0–217.5)	168.0 (72.0–317.0)	0.005
Post-operative CVVHDF^a^	138 (20.1)	41 (12.9)	97 (26.4)	<0.001
Post-operative LCOS^a^	187 (26.0)	41 (12.9)	146 (39.7)	<0.001
Post-operative cerebral event^a^	50 (6.9)	18 (5.7)	32 (8.7)	0.127
Discharge POD^b^	15.0 (10.0–26.0)	15.0 (10.0–23.0)	17.0 (10.0–30.0)	0.167
Post-operative LVEF^a^ (%)	45.0 (37.5–50.0)	45.0 (40.0–50.0)	41.5 (35.0–48.0)	0.018

Values are median (interquartile range) or *n* (%).

VSR, ventricular septal rupture; LVFWR, left ventricular free-wall rupture; PMR, papillary muscle rupture; AMI, acute myocardial infarction; MC, mechanical complication; MV, mitral valve; CPB, cardiopulmonary bypass; ACC, aortic cross-clamping; CABG, coronary artery bypass grafting; IABP, intra-aortic balloon pump; ECMO, extracorporeal membrane oxygenation; h, hours; CVVHDF, continuous veno-venous haemodiafiltration; LCOS, low-cardiac output syndrome; POD, post-operative day; LVEF, left ventricular ejection fraction.

a Operative survivors (*n* = 686).

b Hospital survivors (*n* = 451).

c Type of rupture and repair technique or procedure were calculated for patients with LVFWR (*n* = 150), VSR (*n* = 451), and PMR (*n* = 153), respectively.

Post-operatively, inotropic agents were used in 80.0% of subjects, and mechanical circulatory support (MCS) devices were adopted in almost two-thirds of patients. Eighty-two subjects required re-thoracotomy for mediastinal bleeding, whereas reoperation for other reasons was necessary in 9.0% of cases. Median intensive care unit stay was 6.0 (IQR: 3.0–11.3) days, while total post-operative hospitalization lasted about 2 weeks.

### In-hospital outcomes

The vast majority of patients experienced a complicated post-operative course, mostly cardiac-related. Indeed, about one-third of subjects developed low-cardiac output syndrome (LCOS) or some form of ventricular dysfunction after surgery. Recurrent rupture occurred in 11.4% of subjects (mostly VSR-related), and a reintervention was required in approximately half of the cases. The list of major postoperative complications is presented in *Table 3*.

**Table 3:** Outcomes and causes of in-hospital death

Variable	Patients (*n* = 720)
Intraoperative mortality	34 (4.7)
In-hospital mortality	269 (37.4)
Causes of death^a^	
On-table death	34 (12.6)
LCOS	94 (34.9)
MOF	46 (17.1)
Re-rupture	20 (7.4)
Bowel infarction	10 (3.7)
CVA	13 (4.8)
Sepsis/mediastinitis/pneumonia	20 (7.4)
AMI	6 (2.2)
AKI	9 (3.3)
Other	7 (2.6)
Unknown	10 (3.7)
Main post-operative complications^b^	
Cardiac	
LCOS/ventricular failure	224 (32.7)
Re-rupture	78 (11.4)
Atrial fibrillation	109 (15.9)
PM implantation	15 (2.2)
AMI	16 (2.3)
Cardiac tamponade	19 (2.8)
Pulmonary	
Pneumonia	71 (10.3)
ARDS	35 (5.1)
Infectious	
Sepsis	77 (11.2)
Mediastinitis	9 (1.3)
Urinary tract infection	13 (1.9)
Renal	
AKI	202 (29.4)
Dialysis	138 (20.1)
Neurological	
Stroke	42 (6.1)
Intracranial haemorrhage	10 (1.5)
Delirium	31 (4.5)
Gastrointestinal	
Bowel infarction	12 (1.7)
Bleeding	1 (0.1)
Late mortality^c^	133 (29.5)
Overall mortality	402 (55.8)

Values are *n* (%).

LCOS, low cardiac output syndrome; MOF, multiorgan failure; CVA, cerebrovascular accident; AMI, acute myocardial infarction; AKI, acute kidney injury; PM, pacemaker; ARDS, acute respiratory distress syndrome.

a Among 269 patients who died in-hospital.

b Among 686 patients who survived surgery.

c Among 451 patients who survived to hospital discharge.

Intraoperative mortality was recorded in 34 patients (4.7%); almost 60% of these deaths were due to severe univentricular or biventricular dysfunction precluding CPB weaning, while the remaining were related to the impossibility of repairing the rupture or to uncontrollable bleeding.

In-hospital mortality rate was 37.4% (*n* = 269) and, according to different types of MCs, it was as follows: 51.5% for multiple MCs, 41.1% for VSR, 35.8% for LVFWR, and 23.8% for PMR (*P* = 0.001). In more than 50% of cases, the cause of early death was ventricular dysfunction or any of its consequences (i.e. LCOS, multiorgan failure). Recurrent rupture represented the cause of death in 20 subjects, equally distributed between VSR and LVFWR (*Table 3*).

### Long-term survival

**Figure 2:**
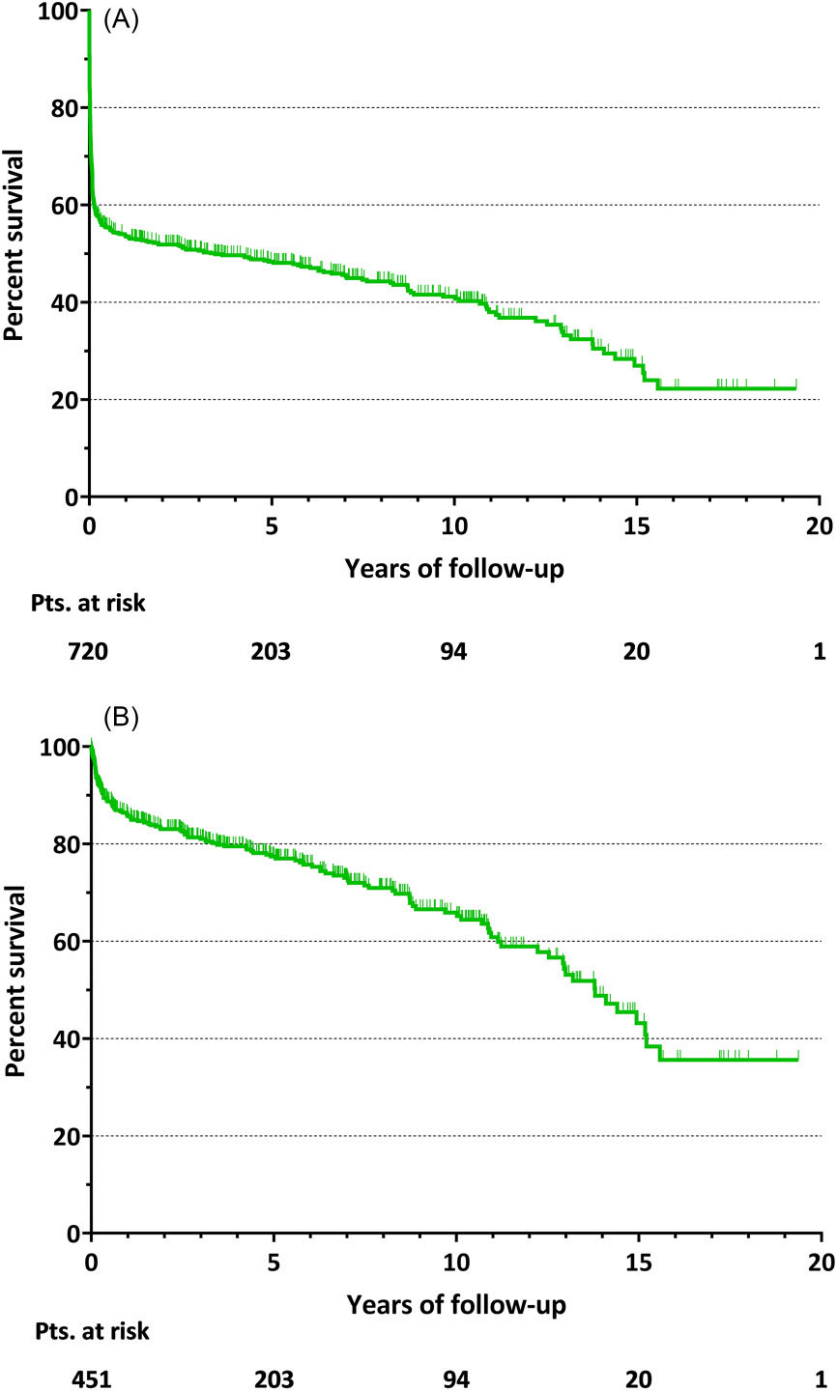
Kaplan–Meier survival curve of cumulative survival for whole population (A) and for hospital survivors (B).

The mean follow-up time for all patients was 3.4 ± 4.6 years. Overall mortality rate was 55.8% (*n* = 402). For the whole population, cumulative survival at 1, 5, and 10 years was 54.0, 48.1, and 41.0%, respectively (*Figure 2A*).

Late mortality was recorded in 133 patients (29.5% of hospital survivors), with a median follow-up of 4.4 (IQR: 1.0–8.6) years. Considering only patients discharged from hospital, cumulative survival at 1, 5, and 10 years was 85.6%, 77.3%, and 65.7%, respectively (*Figure 2B*).

**Figure 3:**
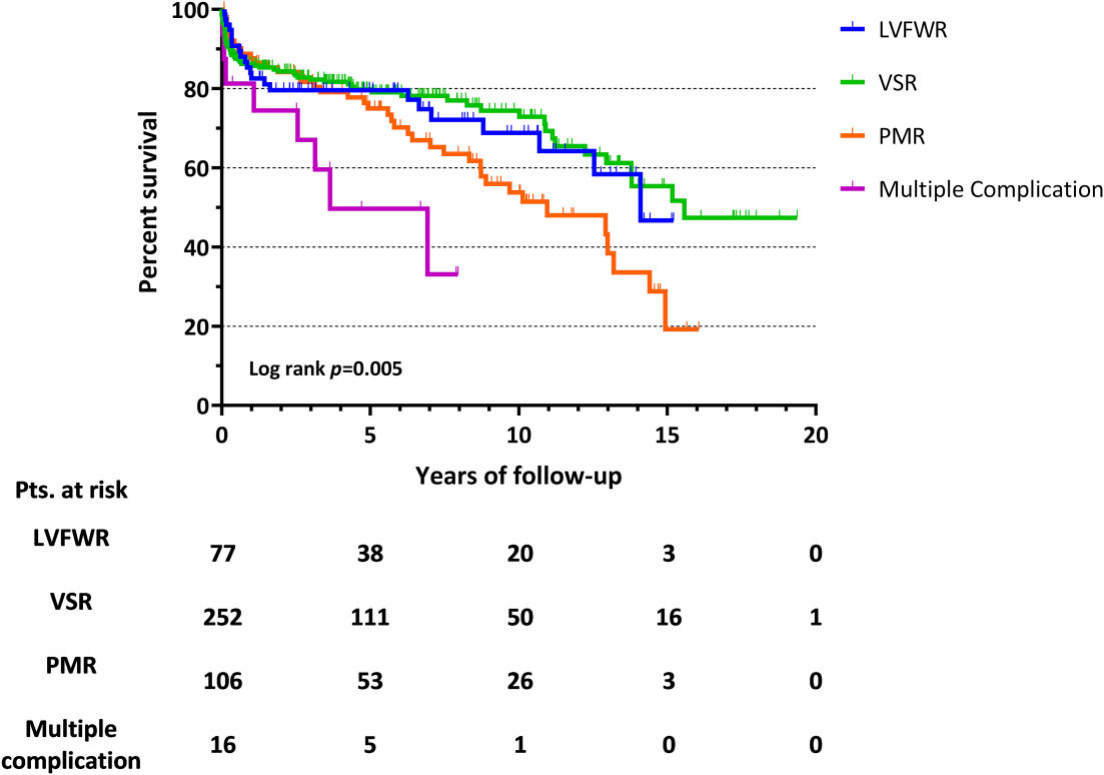
Long-term survival for hospital survivors according to different types of post-infarction mechanical complications. LVFWR, left ventricular free-wall rupture; VSR, ventricular septal rupture; PMR, papillary muscle rupture; AMI, acute myocardial infarction; MCs, mechanical complications.

*Figure 3* depicts the late survival according to different types of post-AMI MCs, showing a significantly higher mortality for subjects with multiple MCs (log-rank *P* = 0.005), followed by PMR patients. More specifically, 1, 5, and 10 year survivals for hospital survivors were as follows: 84.0, 79.6, and 68.8% for LVFWR; 86.2, 80.2, and 74.4% for VSR; 89.0, 75.2, and 53.6% for PMR. A pairwise comparison showed that, among individuals with single MC, subjects operated for VSR had a significantly better long-term survival than PMR patients (log-rank *P* = 0.022).

### Predictors of mortality

**Figure 4:**
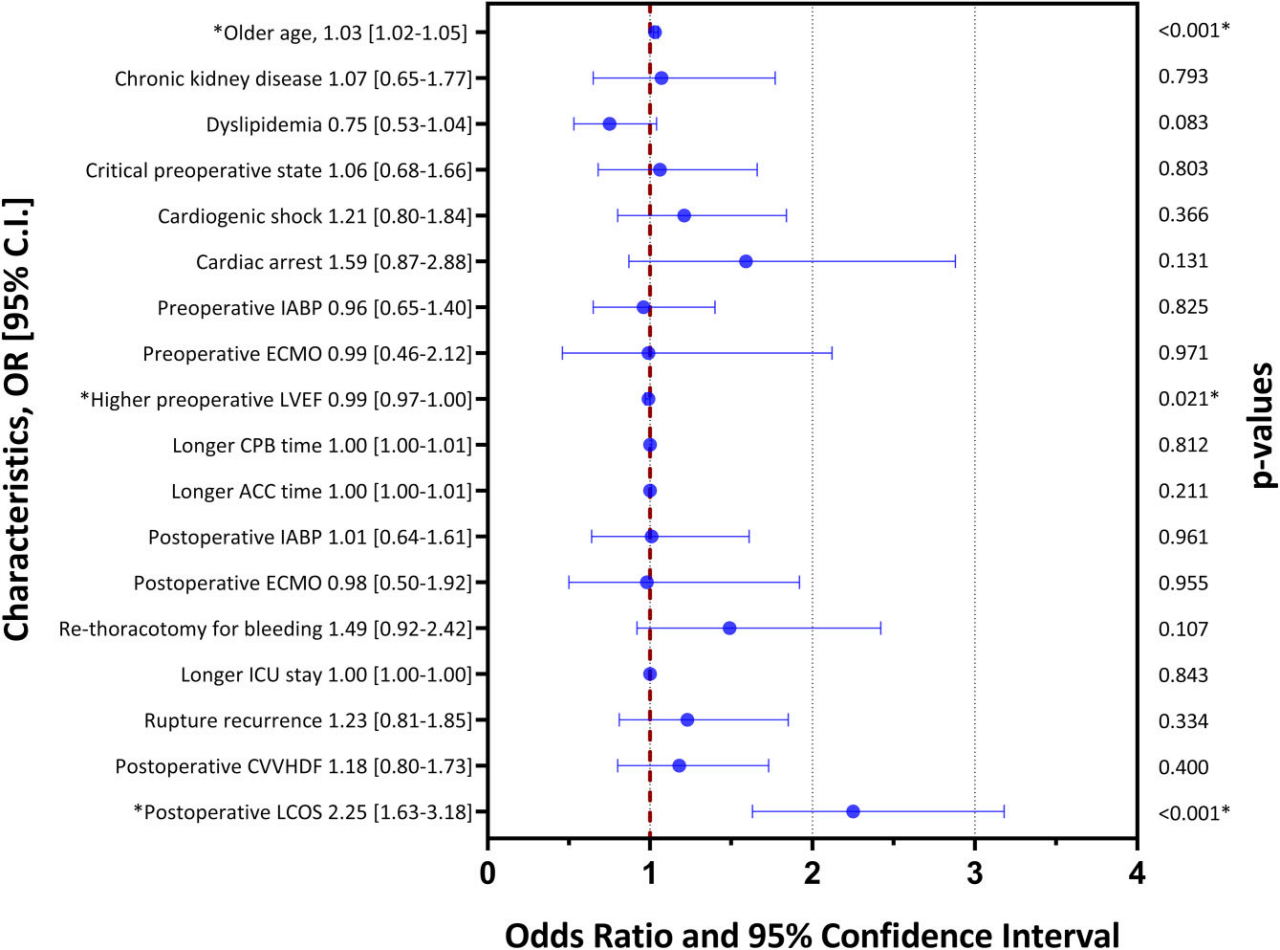
Predictors of overall mortality among patients with post-infarction mechanical complications underwent surgical treatment. IABP, intra-aortic balloon pump; ECMO, extracorporeal membrane oxygenation; LVEF, left ventricular ejection fraction; CPB, cardiopulmonary bypass; ACC, aortic cross-clamp; ICU, intensive care unit; CVVHD, continuous veno-venous haemodiafiltration; LCOS, low cardiac output syndrome.

Results of multivariable regression analysis for overall mortality are outlined in *Figure 4*. Independent predictors of overall mortality included older age (HR: 1.03; 95% CI: 1.02–1.05; *P* < 0.001) and the development of postoperative LCOS (HR: 2.25; 95% CI: 1.63–3.18; *P* < 0.001). Higher preoperative left ventricular ejection fraction (LVEF) on admission predicted better overall survival (HR: 0.99; 95% CI: 0.97–1.00; *P* = 0.021).

**Figure 5:**
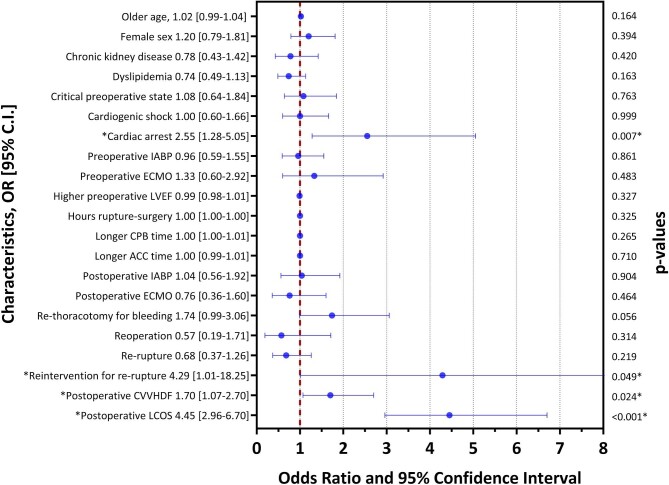
Predictors of early mortality among patients with post-infarction mechanical complications underwent surgical treatment. IABP, intra-aortic balloon pump; ECMO, extracorporeal membrane oxygenation; LVEF, left ventricular ejection fraction; CPB, cardiopulmonary bypass; ACC, aortic cross-clamp; CVVHD, continuous veno-venous haemodiafiltration; LCOS, low cardiac output syndrome.

Independent predictors of early mortality are shown in *Figure 5*. Multivariable analysis identified cardiac arrest on admission (HR: 2.55; 95% CI: 1.28–5.05; *P* = 0.007), reintervention for recurrent rupture (HR: 4.29; 95% CI: 1.01–18.25; *P* = 0.049), post-operative continuous veno-venous haemodiafiltration (CVVHD) (HR: 1.70; 95% CI: 1.07–2.70; *P* = 0.024), and post-operative LCOS (HR: 4.45; 95% CI: 2.96–6.70; *P* < 0.001) as factors independently associated with in-hospital mortality.

**Figure 6:**
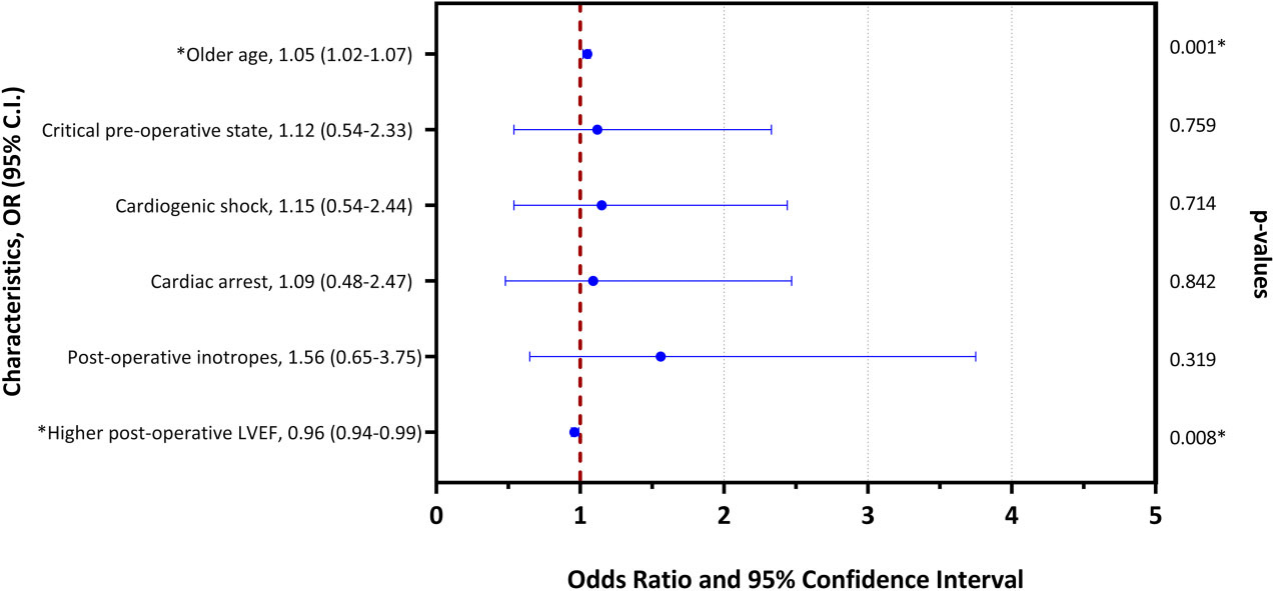
Predictors of late mortality for hospital survivors. LVEF, left ventricular ejection fraction.

**Figure 7:**
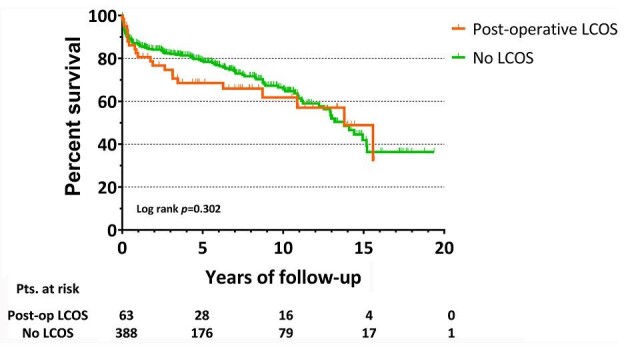
Long-term survival for hospital survivors according to post-operative low cardiac output syndrome. LCOS, low cardiac output syndrome; pts, patients; post-op, post-operative.

For patients who were discharged from the hospital (*n* = 451), older age emerged as an independent predictor of mortality at follow-up (HR: 1.05; 95% CI: 1.02–1.07; *P* = 0.001), while higher post-operative LVEF predicted long-term survival (HR: 0.96; 95% CI: 0.94–0.99; *P* = 0.008), as depicted in *Figure 6*. On the other hand, *Figure 7* shows that no difference was found in late survival between patients who experienced post-operative LCOS vs. those who did not (*P* = 0.302).

## Discussion

In this 19-year observational analysis of more than 700 patients who developed MCs following AMI and who underwent appropriate surgical treatment, we evaluated outcomes and long-term survival in the contemporary era. Our major findings include the following: (i) the in-hospital mortality rate was 37.4%; (ii) cardiac arrest on admission, reintervention for recurrent rupture, postoperative LCOS and need for CVVHD were independently associated with early death; (iii) cumulative 5- and 10-year survival was 77.3 and 65.7% for subjects discharged alive from hospital, respectively; (iv) late survival was higher for VSR patients, fully inverting the trend of in-hospital mortality; (v) older age and post-operative LCOS predicted overall mortality, while higher pre-operative LVEF was linked to better survival; and (vi) while older age remained also predictor of late mortality for patients who survived the hospitalization, higher post-operative LVEF predicted better long-term survival.

Over the past few decades, advances in reperfusion therapies for AMI have resulted in a significant decline in the incidence of MCs.^2,3^ Although studies in the pre-thrombolytic era showed rates as high as 6% with transmural MI,^3^ the incidence was as low as <1% in contemporary trials.^1,2^ Despite such an improvement, the in-hospital mortality rate among patients who developed MCs remains extremely high, even when prompt surgery can be provided, and substantially unchanged over time.^2,3,6^ The present analysis demonstrated that post-AMI MCs still portend a very grim prognosis, with operative mortality approaching 40%. Similar mortality rates were reported by other reports,^1,2^ confirming these post-AMI events to be among the most lethal cardiac surgical diseases.

Cardiac arrest at presentation, reintervention for re-rupture, postoperative kidney injury requiring CVVHD, and LCOS following surgery were found to be the most important predictors for early mortality. In accordance with previous studies and also based on sub-analyses performed on this population, such features are more often encountered in subjects with LVFWR and VSR, who also required significantly higher rates of post-operative IABP and ECMO supports, indicating a higher degree of ventricular dysfunction and its systemic effects in the acute phase, thus possibly explaining the higher rate of in-hospital mortality of these conditions with respect to PMR patients.^7–10^

Recent reports have shown an increased use of MCS in subjects who develop MCs following AMI.^2,11^ In such a scenario, MCS play an important role, not only giving the clinicians time to postpone the definitive treatment but also contributing to improve preoperative patient conditions or, more importantly, to favour myocardial recovery after surgery.^12^ Interestingly, in this study, despite the high number of subjects suffering post-operative LCOS, only 12% of patients received ECMO, indicating a limited use of aggressive MCS. Furthermore, our analysis did not provide evidence to support any benefit of ECMO support on survival. Whether this finding reflects the fact that the use of ECMO is a marker of a sicker cohort of patients who would have faced an otherwise more ominous outcome or simply signals a lack of benefit from the device could not be determined from the present study, also due to the restricted adoption of such MCS in this setting that limits further considerations on its potential benefit. Notwithstanding, looking at the rate of ECMO application and the occurrence of LCOS, a more aggressive approach in patients at risk of developing perioperative LCOS should be considered for timely MCS use, particularly the ones with large preoperative myocardial infarction or reaching surgery in poor clinical conditions.

Given the low incidence of MCs following AMI and the high fatality rate during hospitalization, the literature is particularly focused on the early outcomes, and long-term survival has been less frequently investigated and therefore remains poorly understood, as outlined by a recent meta-analysis by Yousef and colleagues.^13^ Nonetheless, according to the few reports available, outcomes for patients who survive the hospitalization related to the operation appear less ominous than in the acute phase.^8,13–15^ Furthermore, Sulzgruber et al. also suggested that the late survival of subjects facing post-infarction cardiac rupture could approach that of individuals affected by uncomplicated AMI.^16^ The current analysis confirms the excellent life expectancy for hospital survivors, with 5- and 10-year survival of 77.3 and 65.7%, respectively. In light of these implications, surgery should be considered the preferred approach for patients with MCs. Options in subjects who are not candidates for surgery include percutaneous treatment, evaluation for heart transplantation, and palliative medical therapy. However, the definition of patients not suitable for surgical treatment is rather to provide in this context. The presence of a large cardiac injury due to the underlying myocardial necrosis, older patient age and frailty, comorbidities with limited life expectancy, as well as the presence of significant biventricular failure with end-organ impairment certainly deserve a multidisciplinary shock team assessment for the actual potentials and indications for surgical or percutaneous interventions, but also for palliative care referral.

In this study, older age predicted overall mortality, in accordance with previous reports on the topic.^13^ Moreover, the development of LCOS after surgery was an independent predictor of overall morality, whereas preoperative LVEF predicted better survival.

It is noteworthy that, while older age reasonably remained a predictor of late mortality, the abovementioned variables were not independently associated with better survival at follow-up, while higher post-operative LVEF did. Such observation might have a double explanation: first, the overall resulting LV function, once overcome the acute, critical phase, has a reasonable impact on late survival, as shown for the natural history of patients developing post-AMI cardiomyopathy independently from MCs.^17^ Second, although pre-operative LVEF and, especially, post-operative LCOS status strongly drive in-hospital and, consequently, overall mortality, those elements are more relevant in the acute setting. Therefore, it is reasonable to assume that, if such a critical peri-operative phase can be overcome with adequate intensive care management, including the appropriate and less restricted adoption of MCS, these variables do not influence long-term survival. Such considerations underline the pivotal role deserved by optimal peri-operative management in this setting, especially in the most critical patients, not only to improve in-hospital survival but also to provide a benefit that may hopefully last in the long term as well.

A specific mention is deserved by the role of concomitant CABG during time of surgery, especially for its impact on late survival. Indeed, the benefit of revascularizing ischaemic/necrotic myocardial areas during high-risk surgical procedures to potentially protect from the added risk of coronary artery disease (CAD) progression at the expense of increased operative risk and uncertainty advantage remains controversial. A recent meta-analysis showed no difference in both early and late outcomes for patients who underwent concomitant CABG or not.^18^ Similarly, we did not find any survival benefit of simultaneous CABG. We can assume that the real effectiveness of the myocardial revascularization was underestimated by the low number of patients who underwent CABG in our cohort. Indeed, in emergency situations, the execution of coronary angiography is not always possible in order to quickly proceed with surgery. Furthermore, no data regarding multi- or single-vessel CAD, were available and, therefore, no information about complete or incomplete surgical revascularization was present either. In the absence of randomized trials drawing better conclusions it would be advisable to perform CABG when suitable, especially in multivessel coronary disease.^5^

Multiple MCs group brings the highest early and late mortality, reasonably. Considering patients with single MCs, interestingly, while early mortality was highest in VSR and lowest in PMR, this trend inverted during follow-up, where VSR patients showed significantly better survival than PMR ones. The reasons underlying such an unexpected finding should be better investigated. Actually, we can speculate that the contribution of a portion of the septum to the global ventricular function was less relevant for long-term survival, once successfully repaired, than a mitral valve procedure represented mainly by valve replacement without preservation, in most cases, of the sub-valvular apparatus, which might also affect a larger portion of the ventricular free wall and contribute more significantly to impaired post-operative LVEF and worse long-term outcomes.^19,20^ Indeed, mitral regurgitation in the setting of ischemic cardiomyopathy has been traditionally associated with a more unfavourable long-term prognosis, and, therefore, it is reasonable to assume that it may impact negatively also on the late outcome of AMI complicated by cardiac rupture.^21^ Moreover, subgroup analysis revealed that more patients with PMR received concomitant CABG with respect to the other types of MCs. As a matter of fact, we can hypothesize that a more diffused and severe CAD could partially explain our reported observations. Unfortunately, data about the extension of CAD are lacking for these patients, as well as further information about eventual cardiac events and causes of death during follow-up, therefore limiting other possible explanations for this interesting result. Nevertheless, in further sub-analyses, no specific predictors of long-term mortality in PMR patients have been identified, and, in fact, the type of mitral valve procedure (i.e. repair vs. replacement) had no impact on survival at follow-up either. However, such interpretations are mostly speculative and deserve to be better elucidated. Finally, even patients with LVFWR showed a satisfactory late survival, slightly inferior to VSR, and better than PMR patients (albeit not statistically significant). Such new evidence concerning LVFWR further supports the concept that, once overcome the most critical peri-operative phase, even in the more catastrophic events following AMI, the long-term prognosis can be fairly good for these patients.

Multiple gaps remain in the care of MCs of AMI. Due to the very high perioperative mortality, efforts should be directed towards improving preventive and therapeutic measures for high-risk patients with AMI who develop MCs. This entails: more shock team discussion for a timely and appropriate type of MCS, which might include improved preoperative patient conditions; not delayed MCS application in cases of intra-operative cardiac dysfunction and difficulty in weaning from CPB; more frequent use of percutaneous techniques, provided that anatomical features are present, making such an alternative approach feasible; consideration, if applicable, of more advanced treatment (even heart transplantation or durable MCS); more attention to palliative care in cases with prohibitive interventional risks. Further steps in the investigation about post-AMI MCs should also include the subgroup analyses according to different types of MC and to the presence of one or more predictors of unfavourable outcomes in order to stratify patients with different MC types according to their expected risk of mortality and to define specific clinical and therapeutic recommendations for each group. Moreover, a detailed and refined comparison between different types of MCs might provide useful information about their differences and the relative impact on early and late outcomes. All these aspects will represent fields of investigation in order to provide more insights about enhanced patient care or comprehensive evaluation, including contraindications for surgery.

### Study limitations

There are several limitations to this study. First, and most importantly, due to its retrospective nature, both selection bias and unmeasured confounders cannot be excluded. Second, the multicentre design required a data collection form with a limited number of variables to avoid missing data; thus, the possibility that non-reported variables could have influenced the results of the analysis cannot be completely ruled out. Third, the current study did not provide information regarding the durability of surgical repair, and, although reporting late mortality, no sufficient data could be retrieved about the causes of late deaths or re-hospitalizations for cardiac causes at follow-up. Furthermore, only patients with data available about late survival were included in the analysis, albeit representing more than 90% of the whole population collected in the CAUTION study database. Fourth, we evaluated the effect of concomitant CABG on survival; however, we were unable to distinguish the target of revascularization, culprit, or non-culprit vessel.

Finally, since the current registry collects only patients who underwent surgical treatment for post-AMI MCs, no information is available regarding excluded patients who were treated percutaneously or managed conservatively, limiting the comprehensive analysis of survival. Larger multicentre prospective, preferably randomized, trials are required to identify independent predictors of mortality more accurately and to better clarify long-term survival; however, such studies are challenging to conduct and would probably face major ethical issues.

## Conclusions

The present 19-year multicentre analysis confirms that the surgical treatment of MCs following AMI is still associated with high in-hospital mortality, approaching 40%. However, once the initial acute phase has been overcome, surgery for post-infarction MCs restores a promising long-term outcome, with a 10-year survival of 65% for hospital survivors. Late survival is significantly better for VSR patients as compared to PMR patients, inverting the results of early mortality. Such encouraging results emphasize the importance of prompt diagnosis and an aggressive approach for patients developing MCs after AMI. Notably, the survival benefit of simultaneous CABG is uncertain.

## Data availability

The data underlying this article will be shared on reasonable request to the corresponding author.
